# Elevated Preoperative Serum Hs-CRP Level as a Prognostic Factor in Patients Who Underwent Resection for Hepatocellular Carcinoma

**DOI:** 10.1097/MD.0000000000002209

**Published:** 2015-12-11

**Authors:** Yu-Bin Liu, Jie Ying, Su-Juan Kuang, Hao-Sheng Jin, Zi Yin, Liang Chang, Hui Yang, Ying-Liang Ou, Jiang-Hua Zheng, Wei-Dong Zhang, Chuan-Sheng Li, Zhi-Xiang Jian

**Affiliations:** From the Department of Hepatobiliary Surgery, Guangdong General Hospital, Guangdong Academy of Medical Sciences, Guangzhou, P.R. China (Y-BL, S-JK, H-SJ, ZY, LC, HY, Y-LO, J-HZ, Z-XJ) and Department of Infectious, People's Hospital of Xuyi, Jiangsu, P.R. China (JY, W-DZ, C-SL).

## Abstract

To evaluate the effects of preoperative highly sensitive C-reactive protein (Hs-CRP) in serum on the prognostic outcomes of patients with hepatocellular carcinoma (HCC) following hepatic resection in Chinese samples.

From January 2004 to December 2008, a total of 624 consecutive HCC patients who underwent hepatic resection were incorporated. Serum levels of Hs-CRP were tested at preoperation via a collection of venous blood samples. Survival analyses adopted the univariate and multivariate analyses.

In our study, among the 624 screened HCC patients, 516 patients were eventually incorporated and completed follow-up. Positive correlations were found regarding preoperative serum Hs-CRP level and tumor size, Child-Pugh class, or tumor stage (all *P* < 0.0001). Patients with recurrence outcomes and nonsurvivors had increased Hs-CRP levels at preoperation (both *P* < 0.0001). When compared to the Hs-CRP-normal group, the overall survival (OS) and recurrence-free survival rates were evidently decreased in the Hs-CRP-elevated group. Further, preoperative serum Hs-CRP level might be having possible prediction effect regarding survival and recurrence of HCC patients after hepatic section in the multivariate analysis.

Preoperative increased serum Hs-CRP level was an independent prognostic indicator in patients with HCC following hepatic resection in Chinese samples.

## INTRODUCTION

Hepatocellular carcinoma (HCC) accounts for over 500,000 deaths worldwide annually and the overall long-term follow-up results after hepatic section are unsatisfactory.^1^ Besides, HCC accounts for 70% to 85% of primary liver cancer. Due to a relatively higher percentage of chronic hepatitis B carriers, accounting for 10% of its population, China is 1 of the areas with a high prevalence of HCC.^2^ Most patients with HCC are diagnosed at advanced stages of this disease, when curative treatments, such as hepatic resection and liver transplantation, are not feasible.^3^ Currently, multiple curative treatment still have little influence in improving treatment outcomes of those patients, the 5-year recurrence rate is nearly 70%.^4^ In these circumstances, measurable biomarkers identification and earlier detection are pivotal for those patients who were confirmed with HCC.

Chronic inflammation has received multiple attentions in human cancers.^5^ Via inducing cell turnover and under a highly oxidative micro-environment, chronic inflammation is closely associated with local tissue damage, repeated repair, and proliferation, leading to carcinogenesis.^6^ By definition, C-reactive protein (CRP) is an acute phase reactant, which can bind to damaged cells for the activation of the classical complement pathway. The concentration of CRP can increase promptly after an increased amount of synthesis by hepatocytes responding to proinflammatory cytokines, such as interleukin-6. Recent studies have suggested that certain concentration changes in CRP level may contribute harmful prognostic outcomes in patients with various malignancies.^7^ With respect to HCC, previous evidence has proved that preoperative serum CRP level was detected to be increased that might eventually associated with aggressive tumor progression and poor prognostic outcomes in patients with HCC.^7,8^ However, very few studies were focused on the relationship between serum highly sensitive-CRP (Hs-CRP) levels and the prognostic outcomes of HCC in Chinese population. We therefore conducted this study to explore the effects of serum Hs-CRP levels at preoperative on the survival outcomes of patients with HCC following hepatic resection in Chinese samples.

## METHOD

### Ethical Statement

This prospective cross-sectional study involving human subjects was approved by the Institutional Review Board of the Guangdong General Hospital. All patients received written information concerning the background and procedures of the study, and the patients or their relatives gave written informed consent prior to entering the study.

### Patients

From January 2004 to December 2008, a total of 624 consecutive HCC patients who underwent hepatic resection in Guangdong General Hospital were enrolled in this study. Exclusion criteria were listed as follows: patients who received any previous anti-cancer treatment; patients who had other malignancies (n = 20); patients who had systemic infections (n = 13), and died within 30 days after surgery (n = 17); patients who lost to follow-up (n = 32); and patients who failed to provide the informed consent (n = 26). HCC was confirmed histologically diagnosed or based on consistent findings obtained with at least 2 of the following imaging techniques: ultrasonography, computed tomography, and magnetic resonance imaging.

### Clinical Variables

Age, sex, general conditions, pathological parameters as well as clinical biomarkers were all collected. Each patient was classified based on the 3 Child-Pugh's grades.^9^ Besides, according to the modified tumor node metastasis (TNM) staging criteria, tumor staging was established.^10^

### Laboratory Testing

Following an overnight fast, venous blood samples of each subject were taken in the early morning. After 30 min to 2 h, blood samples in the tubes were stored at 20°C for 15 min and centrifuged at 1200g, thereafter, the samples were stored frozen in plastic vials at −80°C for further consecutive analyses. All biomarkers were measured 1 day before tested. Alpha-fetoprotein (AFP) levels were measured with commercially available immunoassay methods (Immulite 2000DPC, Los Angeles, CA) in line with the manufacturer's protocol. Cut-off value was preset into 20 ng/mL (positive: ≥20 ng/mL). Serum Hs-CRP was analyzed by using the Roche Cobas Integra 800 analyzer (Roche Diagnostic, Indianapolis, IN). Inter-assay and intra-assay coefficients of variation (CVs) were 2.3% to 4.2% and 3.1% to 5.5%, respectively, with a preset cut-off value of 0.4 mg/dL. The lower detection limit was 0.03 mg/dL, ranging from 0.03 to 10.0 mg/dL. For all measurements, undetectable levels were preset to have a value equal to the lower limit in the experiment.

### Patient Follow-Up

In the first 2 years, a monthly measurement of AFP was performed. Ultrasonography and dynamic computed tomography were performed every 3 months. These tests were done every 6 months after 2 years. Angiographic examination was done in case of strong suspicion of disease recurrence. The primary endpoint was survival of HCC patients after 5 years from baseline. The secondary endpoint in HCC patients was death within follow-up. For surviving patients, the data were censored on the data of the last follow-up.

### Statistical Analysis

According to the normal or non-normal distribution of data, categorical variables were expressed as percentages were compared using the Chi-squared test. Besides, continuous variables were expressed as means (standard deviation, SD) and medians (interquartile ranges, IQR) for continuous variables, and correlations among continuous variables used Spearman rank-correlation coefficient assessment. Normality was checked by Shapiro–Wilk tests. In contrary, Mann–Whitney *U* test was applied for not normally distributed data comparison between groups, and normally distributed continuous variables analysis was performed using a 2-tailed Student unpaired *t* test. The overall prognostic accuracy of detected biomarkers was summarized by the receiver operating characteristic curves (ROC), with the area under the curve (AUC) acting as a summary measure over criteria and cut-point choices. The relative prognostic importance of serum Hs-CRP levels and other clinical variables on death and recurrence were studied by multivariate analyses using a Cox regression model, where the Cox regression analysis was adopted to obtain crude and multivariate hazard ratios (HR) after adjusted for all significant predictors. The cumulative overall survival (OS) and recurrence-free survival (RFS) were computed by the Kaplan–Meier method and compared by the log-rank test. All statistical calculation was performed with SPSS (version 19.0, SPSS Inc., Chicago, IL). The statistical significance level was *P* < 0.05.

## RESULTS

### Patient Baseline Characteristics

In our study, among the 624 screened eligible HCC patients underwent hepatic resection, 516 patients were finally included and completed follow-up. As shown in Table 1, overall median age was 61 (IQR, 48–69) years and 58.1% were men. Two hundred ninety-nine HCC patients (57.9%) were with liver cirrhosis. At diagnosis, there were 318 (61.6%), 143 (27.7%), and 55 (10.7%) patients classified as Child-Pugh class A, B, and C, respectively. Distribution of tumor stage after TNM (I/II/III/IV) classification was 30.2%/31.7%/25.9%/12.2%, respectively. The median tumor size for the 516 tumors was 4.8 cm (range 2.1–15.5) and the number of patients with solitary tumor was 206 (39.9%). Each case of presence of portal vein thrombosis and extrahepatic metastasis was 112 (21.7%) and 92 (17.8%), respectively. Finally, there were 287 deaths counted in total (HCC recurrence, n = 242 [84.3%], liver failure, n = 10 [3.5%], and other causes, n = 35 [12.2%]) among the 516 cases with completed follow-up information (55.6%).

**TABLE 1:**
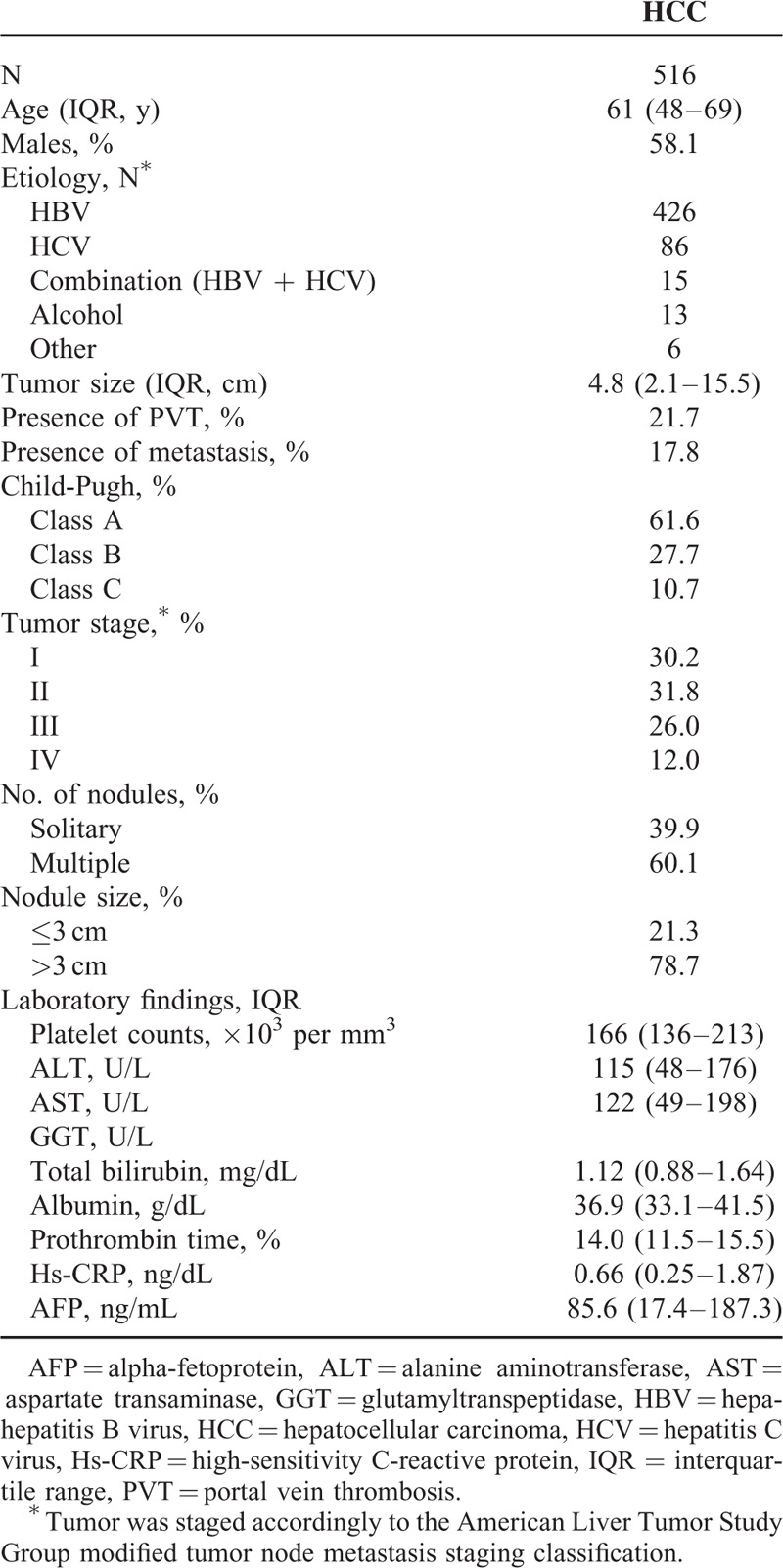
Baseline Characteristics of the Patients With HCC

### Associations Between Baseline Serum Hs-CRP and Patient Characteristics

There was a positive correlation between Hs-CRP and Child-Pugh class (*r* = 0.322, *P* < 0.0001), similar trend was also observed with respect to the correlation between Hs-CRP and tumor stage (*r* = 0.304, *P* < 0.0001). Furthermore, positive correlation regarding levels of Hs-CRP in serum and Child-Pugh class or tumor stage was also found, with significantly statistical difference (*P* = 0.006 and *P* = 0.002, respectively). Besides, the level of Hs-CRP was also elevated as the tumor size increased (*r* = 0.293, *P* < 0.0001). In addition, serum level of Hs-CRP was also found to be positively correlated with the development of venous invasion (*r* = 0.202, *P* = 0.003). However, other factors, such as age, sex, infection time, etiology, HbsAg, hepatitis B virus copies, alanine aminotransferase (ALT), aspartate aminotransferase, total bilirubin, prothrombin time, and AFP were all detected to have none apparent influence regarding serum Hs-CRP level (all *P* > 0.05).

### OS and RFS Rates After Surgery

**FIGURE 1:**
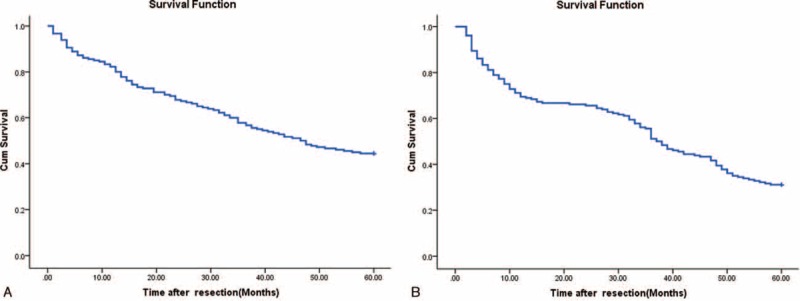
OS and RFS rates of HCC patients after hepatic resection. (A) OS curves in 516 HCC patients after resection; (B) RFS curves in 516 HCC patients after resection. HCC= hepatocellular carcinoma, OS = overall survival, RFS = recurrence-free survival.

**FIGURE 2:**
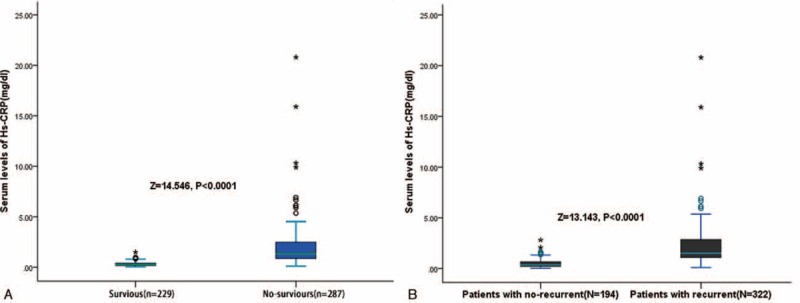
Serum Hs-CRP levels in different groups. (A) Serum levels of Hs-CRP in survivor and nonsurvivor; (B) serum levels of Hs-CRP in patients with recurrence and recurrence-free. Hs-CRP = highly sensitive C-reactive protein.

During the follow-up, 287 (55.6 %) of the 516 patients after surgery had died. The OS rate at 1, 3, and 5 years was 82.2%, 57.8%, and 44.4%, respectively (Fig. 1A). Three hundred twenty-two (62.4%) of 516 patients developed recurrent tumors during follow-up, including 242 patients with intrahepatic recurrence alone, 30 patients with concurrent intrahepatic and extrahepatic recurrences, and 50 patients with extrahepatic recurrence alone. The tumor RFS rates at 1, 3, and 5 years were 69.4%, 51.5%, and 31.3%, respectively (Fig. 1B). Nonsurvivors had significantly higher serum Hs-CRP levels than survivors (1.49 mg/dL [IQR, 1.08–2.86] vs 0.35 mg/dL [IQR, 0.21–0.66]; *P* < 0.0001; Fig. 2A). In the 322 patients with a recurrent outcome, serum Hs-CRP levels was found to have a significant increased tendency than those in patients with no recurrent outcome (1.32 mg/dL [IQR, 0.82–2.49] vs 0.27 mg/dL [IQR, 0.17–0.44]; *P* < 0.0001; Fig. 2B).

### Associations of Baseline Serum Hs-CRP Levels With OS and RFS

The ROC curve illustrated that the optimal cutoff value of serum Hs-CRP levels for OS screening was estimated to be 0.38 mg/dL, yielding a sensitivity and a specificity of 82.5% and 77.4%, respectively, with the AUC at 0.844 (95% confidence interval [CI], 0.765–0.903). Following, with the confirmed value of AUC, Hs-CRP showed a obviously better discriminatory ability as a potential indicator for OS screening of HCC patients when compared with age (AUC, 0.605; 95% CI, 0.548–0.694; *P* < 0.001), AFP (AUC, 0.667; 95% CI, 0.589–0.744; *P* < 0.001), ALT (AUC, 0.594; 95% CI, 0.513–0.674; *P* < 0.001), glutamyltranspeptidase (GGT) (AUC, 0.611; 95% CI, 0.563–0.703; *P* < 0.001), while was in the range of Child-Pugh class (AUC, 0.847; 95% CI, 0.755–0.912; *P* = 0.443). Similarly, the optimal cutoff value of serum Hs-CRP levels for RFS screening was estimated to be 0.41 mg/dL, which yielded a sensitivity of 83.2% and a specificity of 76.2%, with the AUC estimated at 0.851 (95% CI, 0.762–0.911). In this regard, Hs-CRP indicated greater discriminatory power than age (AUC, 0.614; 95% CI, 0.550–0.702; *P* < 0.001), AFP (AUC, 0.676; 95% CI, 0.585–0.752; *P* < 0.001), ALT (AUC, 0.603; 95% CI, 0.522–0.679; *P* < 0.001), GGT (AUC, 0.619; 95% CI, 0.566–0.712; *P* < 0.001), and Child-Pugh class (AUC, 0.757; 95% CI, 0.687–0.834; *P* < 0.05) as well.

**FIGURE 3:**
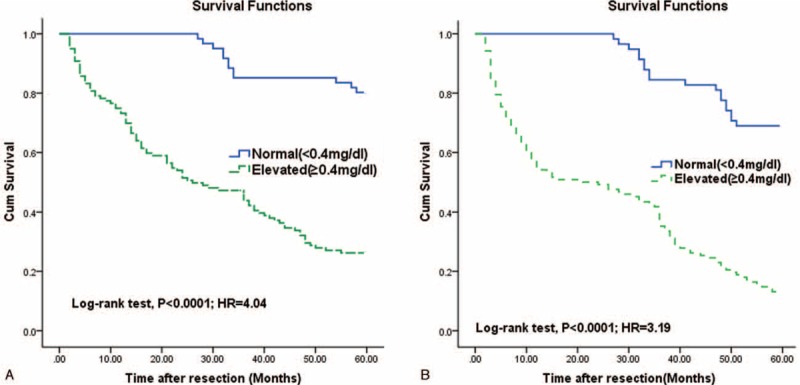
Kaplan–Meier survival based on Hs-CRP levels. OS and RFS rates of HCC patients with high versus low serum Hs-CRP levels. (A) OS rate of HCC patients with high versus low serum Hs-CRP levels. (B) RFS rate of HCC patients with high versus low serum Hs-CRP levels. Patients with high serum Hs-CRP levels had a significantly shorter median OS (31 vs 55 mo; *P* < 0.0001) and RFS (26 vs 53 mo; *P* < 0.0001) than those with low serum Hs-CRP levels. HCC = hepatocellular carcinoma, Hs-CRP = highly sensitive C-reactive protein, OS = overall survival, RFS = recurrence-free survival.

Additionally, the optimal cutoff value of serum Hs-CRP levels for OS and RFS screening were estimated to be 0.38 and 0.41 mg/dL, respectively. In addition, in our laboratory, Hs-CRP levels ≥ 0.4 mg/dL were defined as elevated. Thus, in this study, a cut-off value of 0.4 mg/dL was used. The Kaplan–Meier estimates of OS and RFS stratified by baseline serum Hs-CRP (normal vs elevated) was shown in Figure 3. Patients with elevated serum Hs-CRP had significantly shorter OS and RFS than patients with normal serum Hs-CRP (*P* < 0.0001 and *P* < 0.0001, respectively, log-rank test).

As presented in Table 2, with unadjusted HR of 4.04 (95% CI, 2.59–5.47), there was a close association between Hs-CRP and patients death. After adjusting for all positive predictors, Hs-CRP still remained an independent death predictor with an adjusted HR of 2.85 (95% CI, 1.38–4.01). In addition, age, tumor size, venous invasion, Child-Pugh status, TNM stage, AFP, GGT, ALB, and ALT remained significant death predictors. Similarly, with an unadjusted HR of 3.19 (95% CI, 1.64–4.48), Hs-CRP had a strong association with recurrence. Further, Hs-CRP still have stable predictive ability with an adjusted HR of 2.24 (95% CI, 1.22–3.54) when there was an adjustment for other positive factors. In addition, age, tumor size, venous invasion, Child-Pugh status, TNM stage, AFP, GGT, ALB, and ALT remained significant recurrence predictors.

**TABLE 2:**
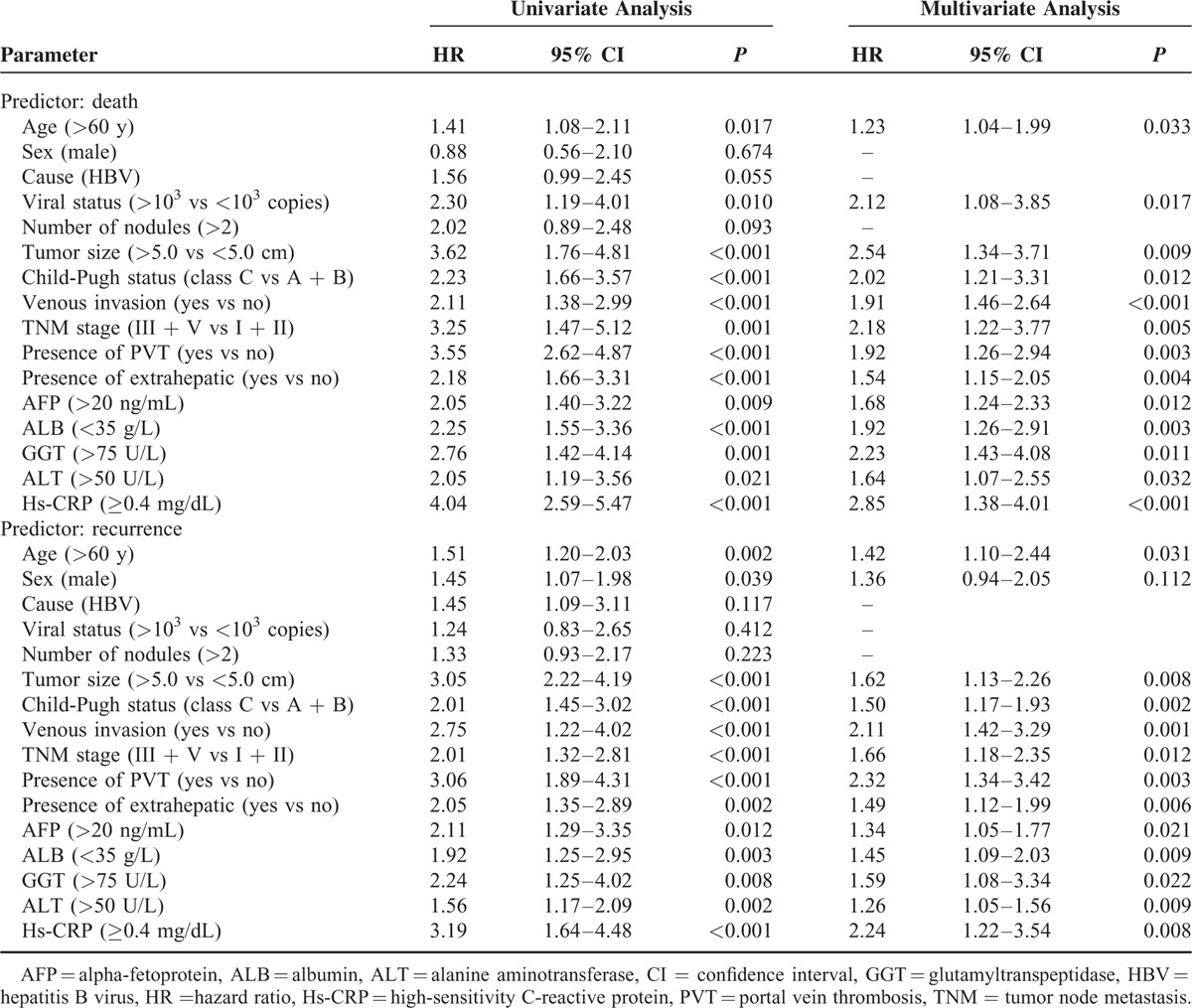
Univariate and Multivariate Analysis for Death and Recurrence by Cox Regression Models

## DISCUSSION

Extensive evidence on the study of HCC has documented that this cancer is a complex and heterogeneous disease, characterized by highly recurrences rates and low 5-year survival rates especially after hepatic resection.^4^ Similarly, we found in our present study that the 5-year survival rate was 44.4%. Many factors, such as vascular invasion of the portal vein and/or hepatic vein and tumor differentiation, are important factors affecting survival and recurrence in HCC cases after a hepatectomy.^11^ Therefore, the risk of metastasis and recurrence prediction would greatly help to guide useful therapeutic treatment choices for HCC patients and therapist.

In fact, multiple-clinical indicators have been found to be strongly related to the prognostic outcomes prediction in patients with HCC.^12–14^ For example, previous studies have suggested that serum YKL-40,^15^ serum hyaluronic acid,^16^ or serum GGT^17^ might be useful for the prediction of HCC prognosis and for OS and RFS prediction in those patients receiving resection or undergoing radiofrequency ablation treatment. In our study, we reported that elevated serum level of Hs-CRP at preoperative was shown to have a positive association with the long-term OS and RFS of HCC patients, suggesting that this biomarker disturbance is prognostically unfavorable, and in turn highlighting the important role of Hs-CRP in predicting the prognostic outcomes in Chinese HCC patients after hepatic resection. Similarly, Oh et al^18^ suggested that CRP was an important prognostic biomarker for HCC, which was in accordance with the results of ours. In addition, pretransplant serum CRP monitoring might also have economic value for predicting prognostic outcomes after liver transplantation for HCC.^19,20^

Inflammation plays an important role in cancer pathogenesis.^21^ Consistent with previous results,^6–8^ our research illustrated that serum level of Hs-CRP at preoperative was an independent survival predictor and was correlated with unfavorable outcomes of HCC. Interestingly, CRP has been suggested as a clinical predictor in many gastrointestinal tumors.^22^ Furthermore, our analysis showed that Hs-CRP level was associated with TNM and Child-Pugh class. Thus, Hs-CRP might become a useful biomarker for HCC prognosis and prediction of treatments efficacy.

There has been not yet clear explanation of how elevated Hs-CRP could be responsible for tumor progression. Possible mechanism speculations were listed as follows. Firstly, main cause of disease recurrence is believed to be intrahepatic metastasis, metachronous or multicentric carcinogenesis.^8^ However, when patients with high serum Hs-CRP levels that may have a chance for tumor cells spreading or metastasis; it cannot be directly detected by either routine imaging or examinations. Importantly, peripheral circulating HCC cells may be critical for the prediction of early recurrence in patients who have high serum CRP levels. Secondly, it has been known that the release of pro-inflammatory cytokines and growth factors, some of which cause metabolic disturbance and decrease in lean tissue, plays some role in this systemic reaction. Tumor growth may be largely promoted with the presence of inflammatory factors, which, in turn, may further have an adverse effect on the systemic inflammatory response.^23^ Moreover, being proved by recent reports, elevated serum CRP levels also have a close relationship with some other prognostic factors, for instance, distant metastasis, tumor size, lymph node metastasis, vascular invasion, and tumor recurrence.^24–26^ Those findings suggested that an elevated serum CRP level could serve as an indicator of the malignant potential of tumors and their poor prognosis.

Certain limitations were existed in the present study. The major limitation of our study was that although patients who showed evidence of inflammatory burden were excluded from the analyses, included participants were likely experiencing some degree of systemic inflammation. As a result, the levels of the inflammatory markers detected here were not solely due to cancer. Secondly, our blood-based measurements of inflammatory markers were taken peripherally, from which we cannot infer local tissue levels. Thirdly, the cross-sectional design of this study was useful in establishing an initial relationship between outcomes and elevated concentrations of CRP preoperative, but subsequent longitudinal studies are necessary to determine the long-term predictive value of Hs-CRP on OS and RFS outcomes. Fourthly, in our study, we did not consider including the Barcelona Clinic Liver Cancer (BCLC) staging systems in our research for categorizing treatment algorithm in this cohort. However, most of the researchers consider BCLC staging as the most useful tool for categorizing treatment recommendation in patients with HCC.^27^ Our study gives a selection bias that is important for affecting our results. In addition, we did not consider evaluating portal hypertension, with either upper endoscopy or hepatic hemodynamic gradient as recommended in other guidelines, which requires further in-depth confirmation of those associations. Lastly, in our study, the patients who died during the first 30 days were excluded in our study. This would cause a significant selection bias and underestimate our findings.

## CONCLUSION

This study eventually confirmed that elevated serum Hs-CRP levels can independently predict poor prognosis for patients with HCC after hepatic resection. Further studies are necessary to confirm this association based on the existence of limitations.
